# Development of an *in silico* prediction system of human renal excretion and clearance from chemical structure information incorporating fraction unbound in plasma as a descriptor

**DOI:** 10.1038/s41598-019-55325-1

**Published:** 2019-12-11

**Authors:** Reiko Watanabe, Rikiya Ohashi, Tsuyoshi Esaki, Hitoshi Kawashima, Yayoi Natsume-Kitatani, Chioko Nagao, Kenji Mizuguchi

**Affiliations:** 1 Laboratory of Bioinformatics, AI Center for Health and Biomedical Research, National Institute of Biomedical Innovation Health and Nutrition, Osaka, Japan; 2 Discovery Technology Laboratories, Mitsubishi Tanabe Pharma Corporation, Saitama, Japan; 3 The Center for Data Science Education and Research, Shiga University, Shiga, Japan; 4 Laboratory of In-silico Drug Design, Center of Drug Design Research, National Institutes of Biomedical Innovation, Health and Nutrition, Osaka, Japan

**Keywords:** Virtual screening, Computational models

## Abstract

Prediction of pharmacokinetic profiles of new chemical entities is essential in drug development to minimize the risks of potential withdrawals. The excretion of unchanged compounds by the kidney constitutes a major route in drug elimination and plays an important role in pharmacokinetics. Herein, we created *in silico* prediction models of the fraction of drug excreted unchanged in the urine (*f*_*e*_) and renal clearance (*CL*_*r*_), with datasets of 411 and 401 compounds using freely available software; notably, all models require chemical structure information alone. The binary classification model for *f*_*e*_ demonstrated a balanced accuracy of 0.74. The two-step prediction system for *CL*_*r*_ was generated using a combination of the classification model to predict excretion-type compounds and regression models to predict the *CL*_*r*_ value for each excretion type. The accuracies of the regression models increased upon adding a descriptor, which was the observed and predicted fraction unbound in plasma (*f*_*u,p*_); 78.6% of the samples in the higher range of renal clearance fell within 2-fold error with predicted *f*_*u,p*_ value. Our prediction system for renal excretion is freely available to the public and can be used as a practical tool for prioritization and optimization of compound synthesis in the early stage of drug discovery.

## Introduction

The excretion process in the urine involves three main processes: glomerular filtration, tubular secretion, and reabsorption^1^. In glomerular filtration, only the unbound drugs in plasma are filtrated and enter the tubular lumen depending on the glomerular filtration rate (GFR) and the extent of the drug fraction unbound in plasma (*f*_*u,p*_). Active tubular secretion is mediated by several transporters for numerous acidic, basic, and some large neutral compounds. A variety of transporters are expressed predominantly in the proximal tubule, executing sequential uptake and efflux that facilitates renal tubular secretion^2^. Reabsorption is mediated by passive diffusion and reuptake by transporters, with the former being especially important for exogenous compounds. Thus, renal excretion is a result of complicated multiple-transport systems, with previous studies reporting that compounds can be classified into reabsorption, intermediate, and secretion type depending on the ratio of renal clearance (*CL*_*r*_) to glomerular filtration^3–5^.

Two important pharmacological indicators in renal drug excretion include the fraction of drug excreted unchanged in urine (*f*_*e*_) and renal clearance (*CL*_*r*_). *f*_*e*_ is an important quantitative indicator showing the contribution of renal excretion for overall drug elimination and *CL*_*r*_ is defined as the proportionality term between urinary excretion rate of unchanged drug and plasma concentration^1^. Predicting the degree of *f*_*e*_ during the drug discovery stage is important to determine the basic principal for the subsequent development stage. Moreover, the use of renal excreted-type drugs should in general be avoided or administered at low dosages for patients with renal failure^6,7^.

The pharmacokinetic profile of a drug is an amalgamation of various properties, such as dissolution, intestinal absorption, plasma protein binding, metabolism, biliary excretion, distribution, and renal excretion. Recently, computer-aided drug design using *in silico* models to predict the absorption, distribution, metabolism, excretion, and toxicity (ADMET) parameters^8–10^ have attracted considerable attention in the field of drug development. This approach is effective to evaluate the physicochemical properties and *in vivo* pharmacokinetics during the early stages of drug discovery. In addition, the use of *in silico* prediction techniques minimizes the expenses and risks of subsequent withdrawals during clinical trials.

Properly validated *in silico* models for ADMET prediction can assist drug design by helping medicinal chemists prioritize suitable lead compounds in the optimization process of early drug discovery. Whereas industrial medicinal chemists may have access to comprehensive commercial suites to predict ADMET properties, this process is difficult for most academic researchers. Alternatively, models built using freely available computational tools can be easily shared with other researchers or can be integrated into other packages. Therefore, such models would constitute valuable assets for both academia and industry.

To the best of our knowledge, no models to predict *f*_*e*_ and *CL*_*r*_ based only on structure information have been developed using freely available software. For the prediction of *f*_*e*_, Doddareddy *et al*.^11^ generated a binary classification model of *f*_*e*_ from structural information calculated using Volsurf and Molconn-Z, with threshold values of *f*_*e*_ set to 0.2 in a dataset containing 130 compounds. This resulted in 65–80% of all test sets to be correctly predicted. Kusama *et al*.^12^ established a binary classification model to predict the major clearance pathways and provided an online prediction system, CPathPred, which was subsequently improved by Toshimoto *et al*.^13^ and Wakayama *et al*.^14^. In the latter prediction model^14^, threshold values of *f*_*e*_ were set to 0.25 for the prediction of renal excretion, yielding an F-measure of 0.67 on the test set for renal excretion with the input of four fundamental parameters (charge, molecular weight [MW], *logD*, and *f*_*u,p*_). To predict the *CL*_*r*_, allometric scaling approaches and *in vitro–in vivo* extrapolation approaches have been extensively utilized. Nevertheless, although allometric scaling is a practical tool, it requires *in vivo CL*_*r*_ data in several animal species, which may be difficult to obtain by academic researchers^15,16^. The *in vitro–in vivo* extrapolation approaches have successfully determined and incorporated *in vitro* permeability data from Caco-2 or LLCPK1 cells into prediction models^17–19^; however, it remains necessary to experimentally determine the individual scaling factors. Furthermore, unique quantitative structure-pharmacokinetics relationships have been constructed to predict the *CL*_*r*_ of drugs or drug-like compounds in humans^20^.

Although the accuracy of previously reported models has been improved^14,20^, such models rely upon either the direct input of experimental values or commercial software for the calculation of descriptors or values of pKa and *log*D. It is difficult to find a free software that can calculate *logD*; moreover, even though ChemAxon (Marvin)^21^ has the ability to calculate pKa on an individual basis, it is not possible to calculate this value for multiple compounds simultaneously using a command line. As it is essential to perform calculations batch-wise when new structures are brought into our prediction system, we could not find suitable free software to calculate *logD* and *pK*_*a*_ for the purpose of this open model.

Previously, we constructed prediction models of the human unbound fraction in plasma (*f*_*u,p*_)^22^, with the *f*_*u,p*_ prediction models released via a freely available tool (*f*_*u,p*_ Predictor, http://adme.nibiohn.go.jp/fup/). As approximately 10% of the blood volume is filtered at the glomerulus by the hydraulic pressure exerted by the arterial blood and, as a general rule, only the unbound drug in plasma is filtered, the value of *f*_*u,p*_ significantly impacts the renal glomerular filtration^23^. Accordingly, Dave *et al*.^20^ pointed out that the *f*_*u,p*_ represents the most important determinant of *CL*_*r*_ prediction. Moreover, *f*_*u,p*_ has been included as one of the four default descriptors in *f*_*e*_ prediction in several reports^12–14^. Thus, we considered that our *f*_*u,p*_ prediction models^22^ might be expanded to predict *f*_*e*_ and *CL*_*r*._

Here, we created *f*_*e*_ and *CL*_*r*_ datasets of 411 and 401 compounds, respectively, and generated two types of predictions: 1) binary classification models of *f*_*e*_ and 2) a two-step prediction system of *CL*_*r*_ through a combination of the classification and regression models, incorporating structure information without any experimental values but with predicted *f*_*u,p*_ values, using freely available software. Moreover, the contribution of *f*_*u,p*_ to the accuracy of regression models for *CL*_*r*_ prediction was considered. These *in silico* prediction models are freely available.

## Methods

### Data set preparation and descriptor calculation

The dataset for *f*_*e*_ prediction was acquired from Benet *et al*.^24^ and PharmaPendium^25^. The dataset for *CL*_*r*_ prediction was acquired from the ChEMBL database and the dataset reported by Varma *et al*.^3,26,27^ and Ito *et al*.^5^. Both datasets were created after careful curation to select the values of *f*_*e*_ or *CL*_*r*_ in healthy adult humans for a single administration to obtain higher prediction accuracy^28^. The details of curation are provided in Supplementary Methods.

For the *f*_*e*_, a dataset containing 411 compounds (343 from Benet *et al*.^24,27^ and 68 from PharmaPendium) with *f*_*e*_, *f*_*u,p*_, and structure information was assembled (Dataset*_f*_*e*_). The list of 343 compounds and their *f*_*e*_ values are summarized in Supplementary Table S1; detailed information for the 68 compounds acquired from PharmaPendium has not been presented owing to licensing restrictions.

For the *CL*_*r*_, a dataset containing 401 compounds with experimental *CL*_*r*_ including *f*_*u,p*_ values and structure information was assembled (Dataset*_CL*_*r*_); the clearance ratio (CR)^5^, which is also referred to as the renal extraction ratio^29^, to categorize compounds into three excretion types was calculated using the following equation:

$$CR=CLr/(fu,p\times {\rm{GFR}})$$

The GFR used in this study was 1.8 mL/min/kg (126 mL/min in a 70 Kg man). The compounds were categorized into three types based on their CR. The compounds that displayed CR < 0.67, 0.67 ≤ CR < 1.5, or 1.5 ≤ CR were classified into reabsorption (R) type (net reabsorbed compounds), intermediate (IM) type (apparently not reabsorbed or secreted compounds), and secretion (S) type (net secreted compounds), respectively^5^. Predicted *f*_*u,p*_ was calculated using our previously developed *f*_*u,p*_ predictor^22^. Ionization profiles in the data set were extracted from the ChEMBL database.

We employed the open source programs Mordred (ver. 1.0.0)^30^ and PaDEL-Descriptor^31^ to calculate the two-dimensional (2D) descriptors and fingerprints (Extended, KlekotaRoth, and AtomPairs2D), respectively. *LogD*pH7.4 and *pK*_*a*_ (apKa) values were calculated using ChemAxon calculator plugin software (Budapest, Hungary) because of the importance of *LogD* and *pK*_*a*_ as pharmacokinetic parameters; these values were used only for visualizing the chemical space by principal component analysis (PCA).

### Data analysis

Data analysis was performed in R (version 3.5.1^32^), and the results were visualised using the ggplot2^33^ and ggfortify^34^ packages. In total, 11 descriptors, i.e., MW, topological polar surface area, SLogP, LogD pH 7.4, apKa, bpKa, hydrogen bond acceptor (HBAcc), hydrogen bond donor (HBDon), number of aromatic atoms (nAromAtom), number of aromatic bonds (nAromBond), and the number of rotatable bonds (nRot), were used for PCA.

### Processes of model construction

The caret^35^ package in R was used to build the prediction models. An overview of the common process in model construction is shown in Supplementary Scheme S1. The data sets were split into training and test sets using random selection at a ratio of 8:2. In the training set, descriptors that showed near-zero-variance and absolute correlations >0.90 were identified and excluded by calculating the frequency ratio using the nearZeroVar function and by creating a correlation matrix using the findCorrelation function in the caret package. Thereafter, descriptors that significantly contributed to the prediction accuracy were selected using the Boruta^36^ algorithm to automatically rank and omit descriptors based on the random forest (RF) classification algorithm with the training set. Boruta is a wrapper built around the RF classification algorithm implemented in the R package randomForest^37^, which provides unbiased and stable selection of important and non-important attributes.

Prediction models were constructed using various machine learning techniques including linear and non-linear methods; i.e., RF, support vector machine (SVM with radial functions), artificial neural network (ANN), and partial least squares (PLS), to obtain the most accurate model for our data set. To adopt each technique, the train function was passed with method parameters set as rf, svm, nnet, and pls in the caret package. We used the automatic grid search of each tuning parameter with four (tuneLength = 4) values of each in the caret package to prioritize the optimal parameters for our predictions and models were created using a 10-fold cross validation. For 3-class classification, the RF algorithms can naturally handle multiclass classification, whereas all-versus-all and all-versus-rest approaches were used for multiclass SVM in the e1071 package^38^ and multinomial log-linear models via neural networks in the nnet package^39^, respectively. The generated models were evaluated with the test set. Kappa (True accuracy), balanced accuracy, sensitivity, and specificity obtained from the confusion matrix in classification models, and r-squared (r^2^, coefficient of determination) and root mean squared error (RMSE) in regression models were used to evaluate their performance on the test set. The best models were chosen according to the value of Kappa or r^2^ of the test set in the classification and regression model, respectively.

### Model construction for *f*_*e*_ and *CL*_*r*_ prediction

As descriptors, more than 1600 2D descriptors calculated via Mordred and 5640 Extended, KlekotaRoth, and AtomPairs2D fingerprints generated using PaDEL-Descriptor were prepared, and descriptors for which the calculation failed were excluded (Supplementary Information 3). The 6974 and 6976 descriptors in *f*_*e*_ and *CL*_*r*_ prediction models were initially used for model construction and descriptors selected using the Boruta^36^ algorithm were finally applied for the predictions. Dataset_*f*_*e*_ was split into 328 and 83 compounds for training and test sets, respectively, using random selection and the prediction model was constructed. Dataset_*CL*_*r*_ containing 401 compounds was split by random selection at a 1:9 ratio into 41 and 360 compounds to isolate the external test set. Thereafter, the other 360 compounds were split at 8:2 (278 and 72 compounds) for 3-class classification models; in parallel, the other 360 compounds were classified into three excretion types; 94 reabsorption (R), 86 intermediate (IM), and 180 secretion (S) type compounds according to their CR calculated using *CL*_*r*_, *f*_*u,p*_, and GFR values. Subsets were defined as Dataset_*CL*_*r*__R, Dataset_*CL*_*r*__IM, and Dataset_*CL*_*r*__S, respectively. An overview of *CL*_*r*_ model construction is shown in Supplementary Scheme S2.

## Results

### Distribution and chemical space analysis in Dataset*_f*_*e*_ and Dataset*_CL*_*r*_

**Figure 1:**
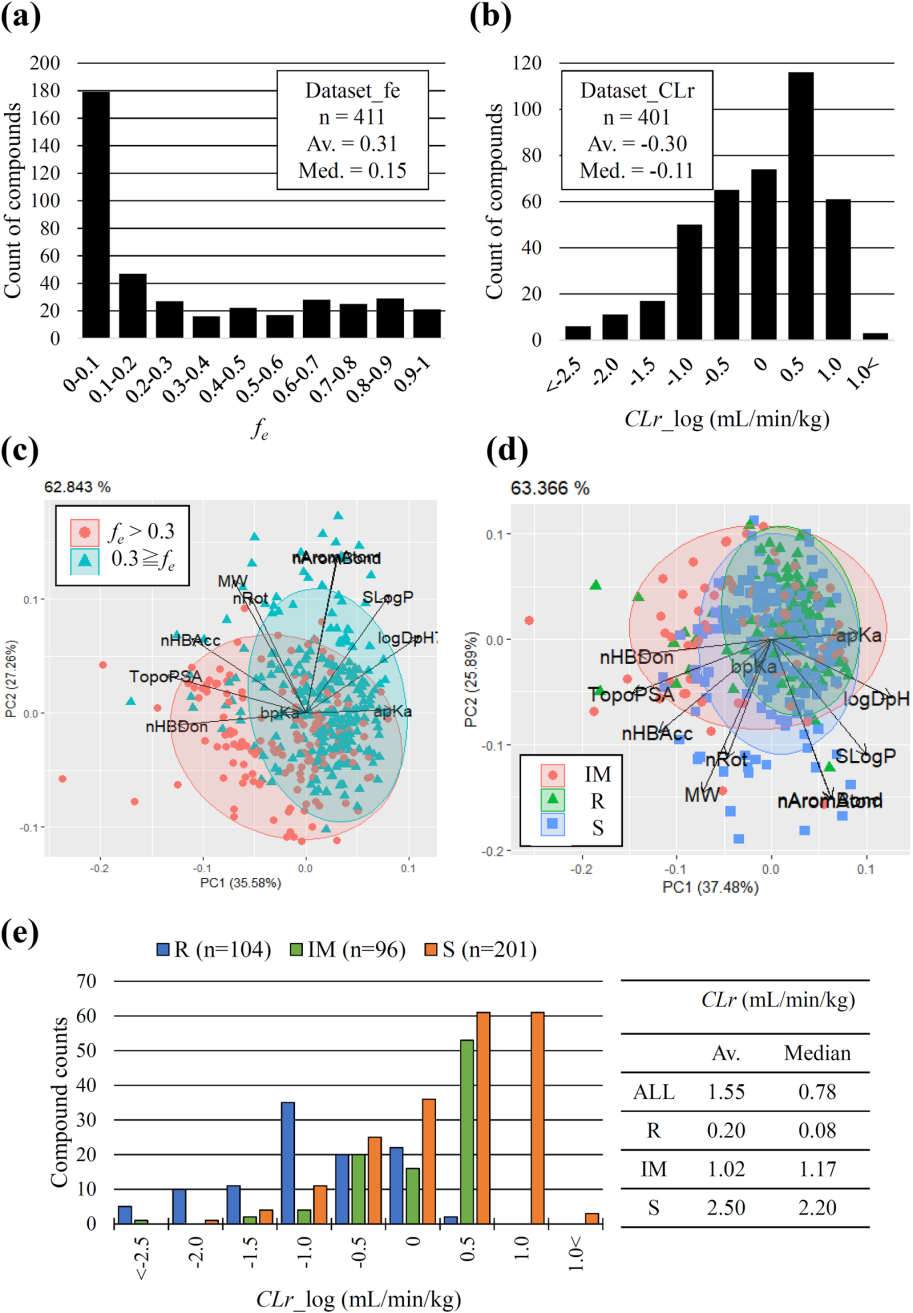
(**a**) Distribution of *f*_*e*_ in Dataset_*f*_*e*_ consisting of 411 compounds. Average and median are shown in the top-right. (**b**) Distribution of *CL*_*r*_ with logarithmic scale in Dataset_*CL*_*r*_ consisting of 401 compounds. Average and median are shown in the top left. (**c**) The chemical space of Dataset_*f*_*e*_ with classification by the threshold set to 0.30. The frames indicate 95% normal confidence ellipses in the assembled 411 compounds with *f*_*e*_ ≥ 0.3 (red) and *f*_*e*_ < 0.3 (green). (**d**) The chemical space of Dataset_*CL*_*r*_ in 96 inte_*r*_mediate (IM, red circle), 104 reabsorption (R, green triangle), and 201 secretion (S, blue square) types. (**e**) Plot of compound counts depending on CR type. Average and median of *CL*_*r*_ in each CR type are shown on the right.

Dataset*_f*_*e*_ and Dataset*_CL*_*r*_, consisting of 411 and 401 compounds, respectively, were weighted towards the lower range of *f*_*e*_ and *CL*_*r*_, with 220 compounds that were overlapped. Distribution of *f*_*e*_ in Dataset_*f*_*e*_ and *CL*_*r*_ with a logarithmic scale in Dataset*_CL*_*r*_ are shown in Fig. 1a,b, and that of *CL*_*r*_ in the original scale is shown in Supplementary Fig. S1; this characteristic was also observed regarding the data sets used in previous reports^11,20^. The chemical spaces of the two datasets were visualized by PCA along with classification, with the threshold set to 0.30 in Dataset_*f*_*e*_ (Fig. 1c) and with CR types such as R, IM, and S in Dataset*_CL*_*r*_ (Fig. 1d). A total of 11 descriptors, all of which are generally considered to be important parameters for synthetic expansion, were used for the analysis. Compounds with higher *f*_*e*_ were less lipophilic than those with lower *f*_*e*_, reflecting the fact that water soluble drugs generally undergo renal excretion. In Dataset_*CL*_*r*_, most of chemical space in R, IM and S type were overlapped, and it was difficult to separate the three classes using these 11 descriptors, indicating that R, IM, S compounds have similar physicochemical properties (Fig. 1d). It was considered reasonable that R type compounds showed a lower *CL*_*r*,_ S type compounds showed a higher *CL*_*r*_ and IM type compounds showed medium *CL*_*r*_ (Fig. 1e). The averages of *CL*_*r*_ were 0.20, 1.02, and 2.50 mL/min/kg in R, IM, and S types, respectively. The relationship between *f*_*e*_ and *CL*_*r*_ or observed *f*_*u,p*_ in logarithmic scale, depends on the ionization properties of the compounds, was also analysed. No trend existed in the distribution of *CLr* in each ionization property and the assembled data set spanned a chemical space similar to that of the approved drugs (Supplementary Fig. S2).

### Classification models to predict the extent of *f*_*e*_

Binary classification models were created with *f*_*e*_ threshold value set to 0.30 to define the low and high/medium classes, with 158 and 253 compounds classified into the high/medium and low class, respectively. These thresholds were chosen according to previous reports^2,14^. Fifty one descriptors were finally selected in the training set using the Boruta algorithm^36^. Prediction models were trained in a training set comprising 328 compounds, to which four machine learning methods (RF, SVM with radial, ANN, and PLS) were applied. Each model was validated on the common test set containing 83 compounds; the statistical results of the models are summarized in Table 1. Kappa was 0.46–0.52 and 0.29–0.49 in the training and test set, respectively. Balanced accuracy and specificity, which is the ratio to successfully distinguish the low *f*_*e*_ class, were 0.63–0.74 and 0.76–0.90 in the test set. RF showed the highest Kappa in the test set; RF parameters (ntree and ntry) were 500 and 14, and the model was defined as Model_ *f*_*e*_. In parallel, to evaluate the statistical influence of *f*_*u,p*_ as a descriptor to *f*_*e*_ prediction accuracy, prediction models of *f*_*e*_ were constructed with or without *f*_*u,p*_ values (observed and predicted). Paired t-test analysis revealed no significant difference between the Kappa of Model_ *f*_*e*_ and those of other models with *f*_*u,p*_ (Supplementary Table S2).

**Table 1:** Statistical results of the binary classification models for *f*_*e*_ prediction by each of the four models.

Descriptor	Selected descriptors	Training or Test	Parameter	RF^a^ (Model_—_*fe*)	SVM^a^	ANN^a^	PLS^a^
without *f*_*u,p*_	51	Training	Kappa	0.50	0.46	0.50	0.52
		Test	Kappa	0.49^b^	0.29	0.37	0.38
			Bal. Acc.	0.74	0.63	0.69	0.68
			Sensitivity	0.65	0.39	0.61	0.45
			Specificity	0.84	0.88	0.76	0.90

^a^RF, Random forest; SVM, Support Vector Machine with radial functions; ANN, artificial neural network; PLS, partial least squares.

^b^The highest kappa in the test set among four models.

SLogP was the most important descriptor in all the models, whereas *f*_*u,p*_ was listed as a second important descriptor in the models with *f*_*u,p*_. The top ranked descriptors according to their variable importance for the best models are listed in Supplementary Table S3, and the main important descriptors were common to all the three models including other lipophilic descriptors such as SlogP. In addition, topological descriptors such as ATS (Moreau-Broto autocorrelation), MATS (Moran autocorrelation), GATS (Geary autocorrelation), chi related index (Molecular connectivity), and ETA (Extended topochemical atom) were also determined as important descriptors.

### Relationship between *CL*_*r*_ and *f*_*u,p*_

**Figure 2:**
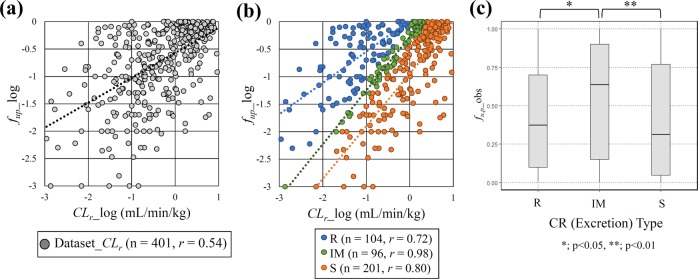
Relationship between *CL*_*r*_ in logarithmic scale and observed *f*_*u,p*_. (**a**) Whole Dataset_*CL*_*r*_ (401 compounds), and (**b**) sub-categorized by CR type (104, 96, and 201 compounds in reabsorption [R], intermediate [IM] and secretion [S] type, respectively). (**c**) Boxplot of observed *f*_*u,p*_ in each excretion type. n; compound counts, *r*; correlation coefficient.

**Figure 3:**
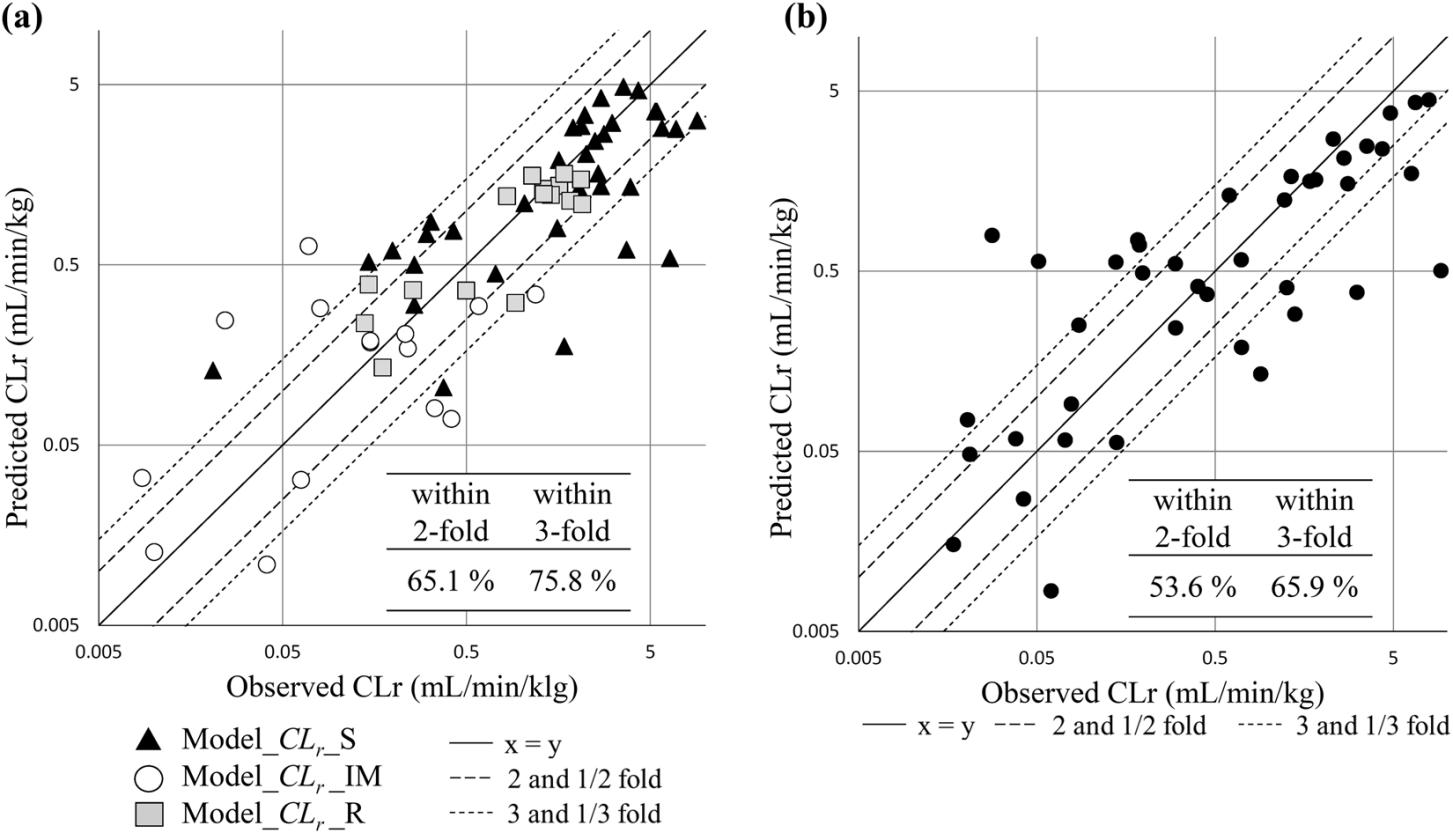
Plot of predicted and observed *CL*_*r*_ by three regression models with predicted *f*_*u,p*_ value. (**a**) in the test set (66 compounds) and (**b**) external test set (41 compounds).

The relationship between *CL*_*r*_ and *f*_*u,p*_ was analysed in Dataset*_CL*_*r*_. The correlation coefficient (*r*) between *CL*_*r*_ and observed *f*_*u,p*_ in logarithmic scale was moderate (*r* = 0.54) (Fig. 2a); however, the correlation between *CL*_*r*_ and observed *f*_*u,p*_ was increased (*r* = 0.72, 0.98, 0.80 in R, IM, and S type, respectively) in the subsets with the CR types (Fig. 3b), suggesting that *f*_*u,p*_ values used as a descriptor are likely effective to create *CL*_*r*_ prediction models in the sub-clustered dataset by CR types. In comparison, the correlation did not change in a subset of Dataset*_CL*_*r*_ with ionization properties (Supplementary Fig. S3). In addition, *f*_*u,p*_ in the IM type was significantly higher than that in the other types (Fig. 2c). This indicated that the mechanism of renal excretion in these compounds is mainly glomerular filtration, with the contribution of secretion by transporters or reabsorption by lipophilicity being low.

Furthermore, upon comparison of the observed and predicted *f*_*u,p*_ values, as shown in Supplementary Fig. S4, a correlation could be seen between observed and predicted *f*_*u,p*_ values (*r = *0.84), with 72.8% and 84.0% of the predicted *f*_*u,p*_ values falling within 2-fold and 3-fold error, respectively. This indicated that the *f*_*u,p*_ predicted by *f*_*u,p*_ predictor^22^ correlated well with the observed *f*_*u,p*_.

### Prediction models for *CL*_*r*_

A comprehensive *CL*_*r*_ prediction model incorporating the whole Dataset*_CL*_*r*_ using several machine learning methods was constructed for a randomly selected training set. This was validated by the test set with or without *f*_*u,p*_ values. Although the average of *r*^2^ appeared to slightly increase (from 0.24 to 0.32) when *f*_*u,p*_ was added as a descriptor, the highest *r*^2^ of all the models was 0.4 in the test set (Supplementary Table S4). As previously reported by Dave *et al*.^20^, a single model was not able to predict the renal clearance of all examined compounds.

As a next step, subsets of Dataset*_CL*_*r*_ by CR type were generated and defined as Dataset*_CL*_*r*__R, Dataset*_CL*_*r*__IM, and Dataset*_CL*_*r*__S as described in the experimental section. Regression models to predict the value of *CL*_*r*_ were generated using four machine learning methods (RF, SVM with radial functions, ANN, and PLS). Three types of descriptors were applied: 1) 6,976 descriptors, 2) 6,976 descriptors + predicted *f*_*u,p*_, and 3) 6,976 descriptors with observed *f*_*u,p*_ in each dataset. The statistical results of each model are summarized in Table 2, and *r*^2^ of the best model and average of *r*^2^ among several models with different randomized split of training and test set are shown. The *p*-values were calculated using the paired *t*-test with *r*^2^ against models without *f*_*u,p*_. All the models showed a significantly higher *r*^2^ when *f*_*u,p*_ values were applied as descriptors: r^2^ in the test set increased from 0.38 to 0.66, 0.56 to 0.92, and 0.41 to 0.62 in the R, IM, and S type, respectively when the observed *f*_*u,p*_ was included as a descriptor, indicating that inclusion of *f*_*u,p*_ values as a descriptor increased the accuracy of the prediction model. In addition, r^2^ in the test set also increased significantly with predicted *f*_*u,p*_ values, and its r^2^ values were slightly lower than those of the models with observed *f*_*u,p*._ In the model with predicted *f*_*u,p*_ values, the PLS in R types and RF in IM and S type showed the best prediction capability, defined as Model*_CL*_*r*__R, Model*_CL*_*r*__IM, and Model*_CL*_*r*__S, respectively. Fold error of the best models are also summarized; the percentage of samples within 2-fold error increased from 37.5% to 56.3% in R type, 68.8% to 100% in IM type, and from 48.6% to 62.9% in S type compounds using the observed *f*_*u,p*_ as a descriptor. The percentage of samples within the 2-fold error also increased with predicted *f*_*u,p*_, as compared with that in the models without *f*_*u,p*_ (to 43.8, 87.5, and 57.1% in R, IM, and S type, respectively). To ensure that this result was not derived from the inclusion of training compounds in the *f*_*u,p*_ prediction model, whose *f*_*u,p*_ can be predicted accurately in general, compounds included in the training set of the *f*_*u,p*_ prediction model were excluded from the test set, with fold errors indicated in parentheses. Although the number of data sets in R type was small and this could accordingly not be compared accurately, a same trend was observed when using the entire data set in IM and S type. Predicted and observed CLr using Model*_CL*_*r*__R, Model*_CL*_*r*__IM, and Model*_CL*_*r*__S in the test set and the external test set containing 41 compounds were plotted in Fig. 3a,b, 75.8% and 65.9% of the compounds fell into within 3-fold error, respectively.

**Table 2:** Statistical results and fold error of the best regression models for *CL*_*r*_ prediction with or without *f*_*u,p*_.

CR type	Descriptor set	Training or Test	The best model				Average	Method^a^
			r^2^	RMSE	Within 2-fold error (%)	Within 3-fold error (%)	r^2^	
Reabsorption Type (R)	Without *f*_*u,p*_	Training	0.48	0.56	—	—	0.50	RF
		Test	0.38	0.61	37.5 (33.3)	43.8 (33.3)	0.23	
	With observed *f*_*u,p*_	Training	0.71	0.44	—	—	0.62*	RF
		Test	0.66	0.46	56.3 (33.3)	62.5 (33.3)	0.53*	
	With predicted *f*_*u,p*_ (Model_*CL*_*r*__R)	Training	0.57	0.51	—	—	0.52*	PLS
		Test	0.52	0.54	43.8 (16.7)	50.0 (33.3)	0.47*	
Intermediate Type (IM)	Without *f*_*u,p*_	Training	0.65	0.38	—	—	0.65	SVM
		Test	0.56	0.28	68.8 (60.0)	93.8 (90.0)	0.43	
	With observed *f*_*u,p*_	Training	0.95	0.17	—	—	0.94*	RF
		Test	0.92	0.12	100 (100)	100 (100)	0.88*	
	With predicted *f*_*u,p*_ (Model_*CL*_*r*__IM)	Training	0.77	0.29	—	—	0.82*	RF
		Test	0.74	0.21	87.5 (83.3)	100 (100)	0.68*	
Secretion Type (S)	Without *f*_*u,p*_	Training	0.43	0.51	—	—	0.46	RF
		Test	0.41	0.46	48.6 (35.0)	68.6 (60.0)	0.36	
	With observed *f*_*u,p*_	Training	0.64	0.39	—	—	0.65*	RF
		Test	0.62	0.37	62.9 (55.0)	80.0 (75.0)	0.57*	
	With predicted *f*_*u,p*_ (Model_*CL*_*r*__S)	Training	0.60	0.42	—	—	0.58*	RF
		Test	0.58	0.40	57.1 (50.0)	80.0 (65.0)	0.46*	

^a^RF, Random forest; SVM, Support Vector Machine with radial functions; PLS, partial least squares; RMSE, root mean squared error. *p-value calculated using the paired t-test with Kappa against model without *f*_*u,p*_ in each CR type (p < 0.05).

The top ranked descriptors according to their variable importance for the three defined best models and a description of those descriptors are summarized in Supplementary Tables S5 and S6. Predicted *f*_*u,p*_ was the most important descriptor in all the models.

To actualize the *CL*_*r*_ prediction using structure information alone, three-class classification models to distinguish CR types (R, IM, and S) were constructed. The statistical results are summarized in Table 3. The RF models showed the highest Kappa (true accuracy) value of 0.32 in the test set, and balanced accuracy of 0.70, 0.58, and 0.68 in R, IM, and S type, respectively, and were defined as Model_*CL*_*r*__CR. Although sensitivity in the R and IM type was not sufficiently high (0.56 and 0.29, respectively), 75% of S type compounds were successfully categorized into the correct type. The other raw parameters are shown in Supplementary Table S7. We also constructed three-class classification models with or without *f*_*u,p*_; no significant difference in the accuracy were detected (Supplementary Table S8).

**Table 3:** Statistical results of the 3-class classification models for *CL*_*r*_ prediction.

Model	Selected descriptors (n)	Training or Test set	Parameter	CR type	RF^a^ (Model_*CL*_*r*__CR)	SVM^a^	ANN^a^	PLS^a^
Without *f*_*u,p*_	15	Training	Kappa	—	0.34	0.34	0.32	0.29
		Test	Kappa	—	0.32^b^	0.19	0.18	0.22
			Sensitivity	R	0.56	0.56	0.56	0.50
				IM	0.29	0.12	0.41	0.18
				S	0.75	0.69	0.47	0.75
			Balanced Accuracy	R	0.7	0.69	0.68	0.68
				IM	0.58	0.50	0.59	0.54
				S	0.68	0.58	0.52	0.59

^a^RF, Random forest; SVM, Support Vector Machine; ANN, artificial neural network; PLS, partial least square.

^b^The highest Kappa shown in the test set.

**Figure 4:**
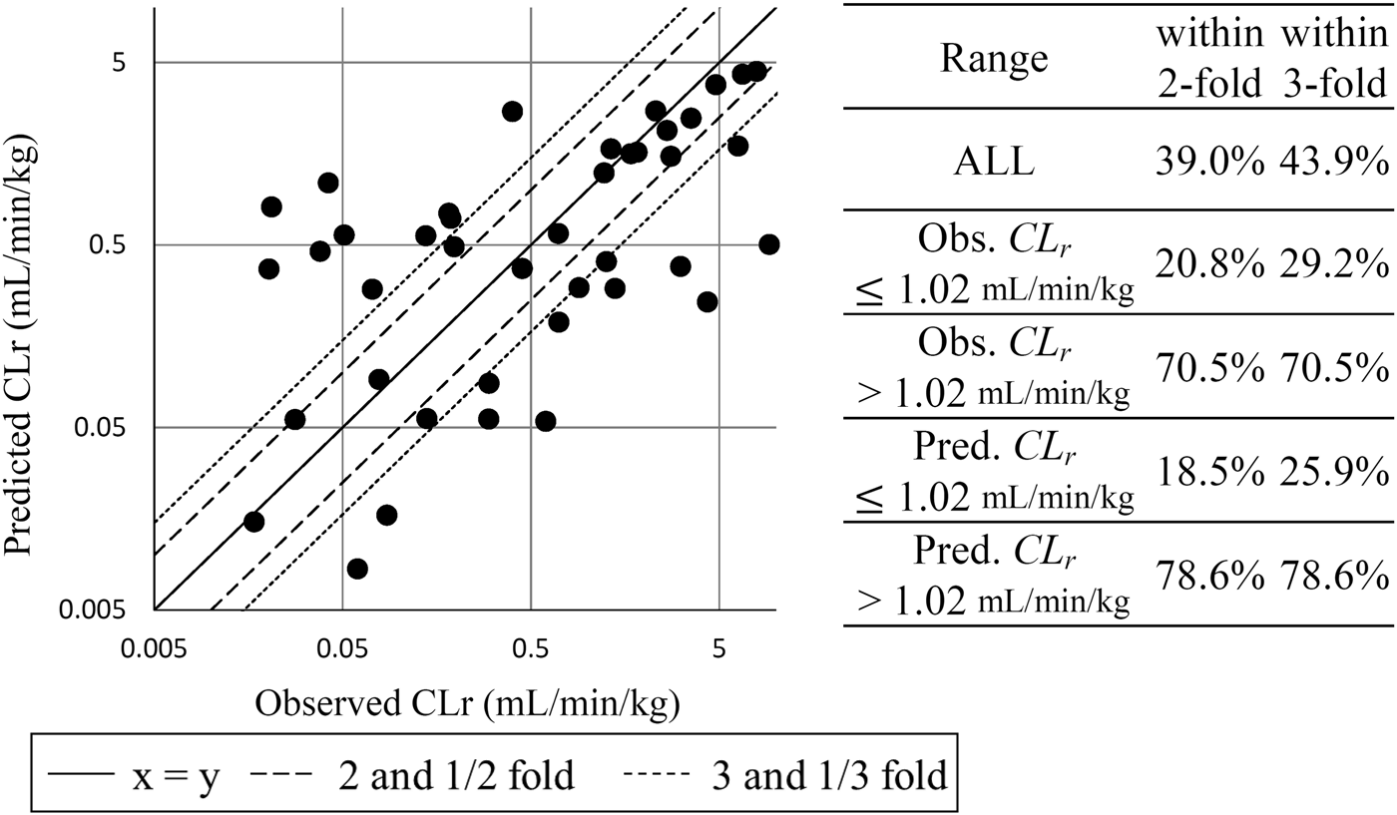
Plot of predicted and observed *CL*_*r*_ in the external validation set consisting of 41 compounds by the two-step prediction system with predicted *f*_*u,p*_ value.

*CL*_*r*_ was predicted with the two-step prediction using *CL*_*r*_ regression models (Model*_CL*_*r*__R, Model*_CL*_*r*__IM, and Model*_CL*_*r*__S) following the prediction of CR type by a three-class classification model (Model*_CL*_*r*__CR). An external test set consisting of 41 compounds that were not included to generate any model was used for the validation. The observed and predicted *CL*_*r*_ values are plotted in Fig. 4; 39.0% and 43.9% of the predicted *CL*_*r*_ values fell into 2- and 3-fold error ranges, respectively. An external validation set was then split into the higher and lower range of observed or predicted *CL*_*r*_ with an average value of *CL*_*r*_ in IM type compounds (*CL*_*r*_ = 1.02 mL/min/kg). When the compounds were split according to observed value of *CL*_*r*_, 70.5% of the compounds fell within 2-fold error in the higher range, and 20.8% and 29.2% of the observed *CL*_*r*_ values fell within 2- and 3-fold error in the lower range of *CL*_*r*_. When the compounds were split by predicted value of *CL*_*r*_, more compounds fell within 2- and 3-fold error in the higher range than in the lower range (78.6% in the higher range and 18.5% and 25.9% in the lower range). Using a combination of the classification model of CR type and the regression model of *CL*_*r*_ in R, IM, and S type, *CL*_*r*_ could be predicted from the structure information using only the freely available software, especially in the higher range of *CL*_*r*_. We also tried two step *CL*_*r*_ prediction models with or without *f*_*u,p*_ and fold error into 2- and 3- fold were not different (Supplementary Table S9).

## Discussion

We developed an *in silico* prediction system to classify compounds into their degree of unchanged excretion in the urine and to predict the value of *CL*_*r*_ using freely available tools without requiring any experimental data. Initially, a binary prediction model of *f*_*e*_ was successfully generated; the threshold was set to 0.30 according to Varma *et al*.^40^, to define the compounds that are well- or poorly-eliminated in the urine. The inclusion of *f*_*u,p*_ did not significantly affect the Kappa in the *f*_*e*_ prediction models; rather, Model_*f*_*e*_ without *f*_*u,p*_ was sufficiently able to predict *f*_*e*_, equivalent to the results of previous studies^11–14^. The majority of the important variables identified in the generated models to predict *f*_*e*_ were common, such that descriptors related to lipophilicity such as SLogP, topological descriptors related to electronic energy, and ionization potential indicators such as AATS, GATS, MATS, and chi comprised the key components of the models. Because lipophilicity is an important determinant for the choice between liver and renal excretion, it is natural that SLogP was the most important descriptor in all the models. In addition, hydrogen bonding interaction descriptors, including ionization potential, total energy, electronic energy, and sum of the total net charge were included in the previously constructed models^4^. Therefore, the inclusion of the descriptors related to lipophilicity, electronic energy, and ionization potential led to the models being able to successfully capture the key factors for *f*_*e*_ prediction. Drug metabolism is generally important as one of the determinants for *f*_*e*_, because the compounds that are well metabolized show smaller values of *f*_*e*_^27,40–42^_._ We believe that it is ideal to predict *f*_*e*_ in consideration of metabolic clearance as a task in future model construction because our *f*_*e*_ prediction model did not take metabolism into consideration; this matter should be addressed in future studies wherein metabolic information has been collected.

**Figure 5:**
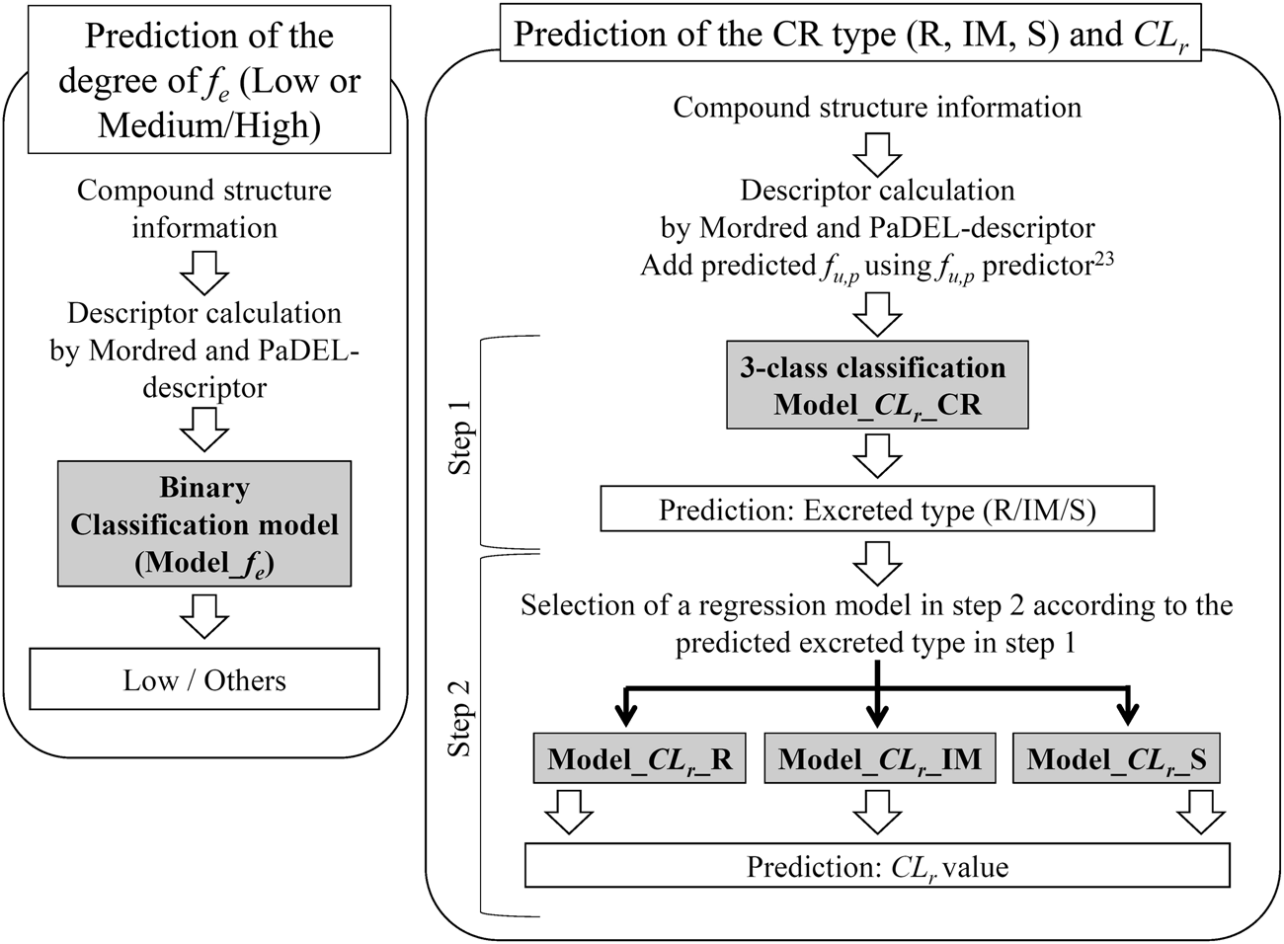
Application of the generated prediction models. Left: *In silico* prediction system for *f*_*e*_ in humans. Right: Two step *in silico* prediction system for *CL*_*r*_ in humans. R; Reabsorption, IM; Intermediate, S; Secretion.

In general, renal impairment alters drug efficacy, often increasing their pharmacological and toxicological effects owing to high concentrations^7^. Moreover, hepatic clearance is known to be impaired in patients with end-stage renal disease because of the accumulation of uremic toxins, which is influenced by the expression of several CYPs^43–45^. Information on renal clearance is useful in the early stages of drug discovery, not only for understanding pharmacokinetic profiles but also for avoiding potential risk in the population with renal impairment, as well as in those with renal disease and advanced age^4^. Our binary model (Model*_f*_*e*_) can be used to screen lead compounds in the early stage of drug discovery (Fig. 5 left). For example, Model*_f*_*e*_ is appropriate for selecting compounds showing low *f*_*e*_ that are not eliminated via the kidney, with an assumption that the drug could be administered to patients with renal impairment.

We concluded that a single *in silico CL*_*r*_ prediction model was unable to predict *CL*_*r*_ even if the *f*_*u,p*_ value was applied as a descriptor, and no discernible linkages between *CL*_*r*_ and ionization property were observed in our study. Comprehensive prediction will be difficult because renal excretion is a result of multiple processes with different mechanisms such as glomerular filtration, secretion, and reabsorption, which are mediated by active transport and passive diffusion by lipophilicity. This interpretation is in accordance with those of Dave *et al*.^20^, who also reported that splitting these compounds according to their ionization property did not improve prediction accuracy of *CL*_*r*_. Dave *et al*.^20^ finally constructed quantitative structure-pharmacokinetics relationships models that could be used to predict *CL*_*r*_ of compounds that (1) undergo net reabsorption, and (2) are substrates and/or inhibitors of human renal transporters. Although the models were accurate, the experimental information, such as class of the compounds in the Biopharmaceutics Drug Disposition Classification System (BDDCS)^24^ and whether those compounds are substrates and/or an inhibitor of renal transporters, is required in advance to determine suitable prediction models. Thus, we aimed to generate a *CL*_*r*_ prediction model in which an external input is not required, using only chemical structure information for devising a practical tool in drug design processes prior to chemical synthesis.

Previously, *f*_*u,p*_ was reported as the most important determinant of renal excretion^5,12,20^. However, the inclusion of *f*_*u,p*_ as a descriptor did not significantly affect *f*_*e*_ and CR type prediction accuracy when the whole dataset was used in this study. In contrast, *r*^2^ of the regression models with the subset of each CR type was significantly increased when observed and predicted *f*_*u,p*_ values were included (Table 2). The results suggest that because of the multiple mechanisms of renal excretion, the impact of *f*_*u,p*_ was observably low in the overall prediction, whereas when Dataset*_CL*_*r*_ was subclustered into three CR types, the influence of *f*_*u,p*_ became more visible among the compounds with similar mechanisms.

The appearance of a drug in the urine is the net result of glomerular filtration, secretion, and reabsorption, for which *CL*_*r*_ is defined as follows:

$$CLr=(1-FR)\,(fu,p\times {\rm{GFR}}+CLs)$$

where *FR* and *CL*_*s*_ are the fractions reabsorbed from the lumen and the secretion clearance, respectively. When the compounds belong to R, IM, and S types, *CL*_*r*_ is expressed by the following respective equations:

$${\rm{Reabsorption}}\,{\rm{type}}\,({\rm{R}}):CLr=(1-{\rm{FR}})\,(fu,p\times {\rm{GFR}})$$

$${\rm{Intermediate}}\,{\rm{type}}\,({\rm{IM}}):CLr=fu,p\times {\rm{GFR}}$$

$${\rm{Secretion}}\,{\rm{type}}\,({\rm{S}}):CLr=fu,p\times {\rm{GFR}}+{\rm{CLs}}$$

All the R, IM, and S type are proportionally affected by *f*_*u,p*_, and *f*_*u,p*_ directly affects the value of *CL*_*r*_ especially in the IM type. On the other hand, *FR* and *CL*_*s*_ can also affect the values of *CL*_*r*_ in addition to *f*_*u,p*_ in the R and S type; information on renal transporters or metabolism related to FR and *CL*_*s*_ is important for *CL*_*r*_ prediction in these types. In addition, when the averages of *r*^2^ in Table 2 were compared, *r*^2^ was increased to the greatest degree in the IM type model (from 0.43 to 0.88 in the test set).

As shown in Figs. 3 and 4, the two-step prediction model of *CL*_*r*_ was generated using a combination of several models. As a first step, the CR type could be predicted using a three-class classification model (Model_*CL*_*r*__CR). As a second step, one of the three regression models (Model*_CL*_*r*__R, Model*_CL*_*r*__IM, or Model*_CL*_*r*__S) was chosen according to the prediction results of Model_*CL*_*r*__CR; then the final values of *CL*_*r*_ were predicted. It should be mentioned that 12 out of 13 compounds that were miss-classified in the first three-class classification did not fall within 3-fold error in the final *CL*_*r*_ prediction, indicating that improved accuracy in step 1 is necessary. Although it was difficult to identify a commonality among miss-classified compounds, cationic charges were frequently included in these miss-classified compounds (Fig. S5). Addition of similar compounds to the dataset or inclusion of *pK*_*a*_ or *log*D information as descriptors which are related to charges will be effective to get higher accuracy. In the present study, we could not include *pK*_*a*_ or *log*D as a descriptor because of the difficulties to find the freely available *pK*_*a*_ or *log*D calculators suitable for our prediction system. Therefore, it is necessary to take into consideration that the accuracy of *CL*_*r*_ prediction is low, particularly when the value of predicted *CL*_*r*_ is <1.02 mL/min/kg. However, in contrast, 78.6% of the compounds in the higher range of predicted *CL*_*r*_ were within 2-fold error, indicating that the results of compounds predicted to be >1.02 mL/min/kg are sufficiently reliable. This can be used for the designing of compounds and subsequent optimization of lead compounds in the early stages of drug discovery (Fig. 4 right).

Our dataset is one of the largest among those previously reported^3,14^. However, several hundreds of compounds were not sufficient to account for all potential diversity. We hope to further expand the number of compounds although it has been difficult to retrieve quality data from the public databases in the present circumstance. It is, therefore, desirable to develop an integrated database with curated data of high quality and sufficient compounds to cover a larger chemical space.

We have developed a prediction system of renal excretion focused on *f*_*e*_ and *CL*_*r*_ based on structure information alone using freely available software, which is available to the public. The prediction of *CL*_*r*_ values from structure information was made possible using a two-step prediction, with three regression models to predict the value of *CL*_*r*_ depending on CR type, following three-class classification into three CR types. Moreover, the accuracies of the regression models were increased by adding observed and predicted *f*_*u,p*_ values, with contribution of *f*_*u,p*_ being the highest in the regression models of IM type. In the external validation set, 78.6% of the samples fell within 2-fold error in the higher range of *CL*_*r*_. These prediction systems of renal excretion are expected to be practical tools, helping medicinal chemists to prioritize the actual synthesis of compounds during the drug design process before synthesis. A new web resource (http://adme.nibiohn.go.jp/renal_ex) has been established to access the online system for the prediction of overall renal excretion, as described in this study.

## Supplementary information

Supplemental_Information_1-3-4

Supplemental_Information_2_Dataset
