# Supplementary material for: Development of an in silico prediction system of human renal excretion and clearance from chemical structure information incorporating fraction unbound in plasma as a descriptor
Source: Sci Rep. 2019 Dec 11;9:18782. doi: 10.1038/s41598-019-55325-1 (PMC6906481; doi:10.1038/s41598-019-55325-1)
Supplement: Supplementary file 1 — Supplemental_Information_1-3-4 [file 41598_2019_55325_MOESM1_ESM.pdf]

Development of an *in silico* prediction system of human renal excretion and clearance from chemical structure information incorporating fraction unbound in plasma as a descriptor

Reiko Watanabe[1,*], Rikiya Ohashi[1,2,*], Tsuyoshi Esaki[1,3], Hitoshi Kawashima[1], Yayoi Natsume-Kitatani[1,4], Chioko Nagao[4] & Kenji Mizuguchi[1,4]

[1]Laboratory of Bioinformatics, AI Center for Health and Biomedical Research, National Institute of Biomedical Innovation Health and Nutrition, Osaka, Japan.
[2]Discovery Technology Laboratories, Mitsubishi Tanabe Pharma Corporation, Saitama, Japan.
[3]The Center for Data Science Education and Research, Shiga University, Shiga, Japan.
[4]Laboratory of In-silico Drug Design, Center of Drug Design Research, National Institutes of Biomedical Innovation, Health and Nutrition, Osaka, Japan.

Supplemental Information 1

Curation process of Datase_*fe*, Dataset_*CLr*, and external validation set

Supplemental Information 3

List of descriptors calculation failed

Supplemental Information 4

Supplemental Schemes S1,2, Tables S1–9, and Figures S1–4

Supplemental Information 1

■ Curation process of Dataset_f_e

Data source: Benet et al.[1] Hosey et al.[2] and PharmaPendium[3]

For Benet et al.[1] -derived data, f_e values of 128 and 627 compounds in human renal excretion via intravenous ($f_{e,i.v.}$) and oral ($f_{e,oral}$) administration were initially collected, respectively. Bioavailability data of 627 compounds via oral administration were searched in the relevant interview forms (Common Technical Document for the registration of Pharmaceuticals) and 252 $f_{e,oral}$ records were corrected according to bioavailability using the following equation: $f_{e,i.v.} = f_{e,oral}/F$. Finally, 380 compounds with f_e value were derived from Benet et al.[1-2]

For PharmaPendium derived data, initially 7213 records of human renal excretion via intravenous administration were initially filtered on the web site (https://www.pharmapendium.com/) and downloaded. Then, 332 records were selected after filtration according to the following criteria.

No.	Action	Column	Word
1	Remove	age	"infant," "newborn baby," "child" and "aged"
2	Select	study group	"healthy" and blank
3	Select	radioactivity	blank
4	Remove	concomitants	If there were other compound names
5	Remove	comment	"pregnant," "patients" or any special notes or abnormalities.

When a single chemical had multiple records, the average values and their standard deviation were calculated. If the standard deviation was more than 0.2, the original sources or the relevant interview forms were checked manually. When this process detected a variation in the condition, the most appropriate values of f_e were selected. Salts were removed by the function to remove small fragments in the ADMET predictor. Finally, 90 compounds were retrieved from PharmaPendium.[3]

The total of 380 and 90 f_e values derived from Benet et al.[1-2] and PharmaPemdium,[3] respectively, were merged into a single dataset (Dataset_f_e). Any overlaps were examined manually using multiple information sources such as standard InChI and the compound name to account for errors in structural representation or orthographical variants, to infer a unified non-redundant dataset. Overlaps were checked using structure information and compound name; if overlapped compounds were identified,

their standard deviation was calculated and the original sources or the relevant interview forms were checked manually. Compounds with molecular weight >1000 Da were removed. f_e values of Memantine, Pravastatin, and Ranitidine were updated according to Hosey et al.[2] Experimental $f_{u,p}$ values were taken from Watanabe et al.,[4] or manually searched in the relevant interview forms. Ionization profiles were extracted from the ChEMBL database. As an additional step, f_e values of four compounds (Memantine, Pravastatin, Ranitidine, and Enalaprilat) were updated according to the report in Hosey et al.[2] Finally, Dataset_ f_e consisting of 411 compounds with f_e, $f_{u,p}$, and structure information was generated.

■ Curation process of Dataset_ CL_r

Data source: ChEMBL[5] and Varma et al. 2009[6], 2010[7], Ito et al.[8], Common Technical Documents (CTD), and five other [9-13] references listed in Supplemental information 2.

For ChEMBL-derived data, the ChEMBL database (ver. 23) was searched with keywords such as "CL_r", "Renal clearance" in standard_type of activity table and "renal clearance" in the description of assay tables, whereby 1090 records were extracted. Then, the records that did not satisfy the inclusion criteria were removed; 827 records were selected after filtration using the following criteria.

No.	Action	Column	Word
1	Remove	unit, value	blank
2	Select	assay_organism	"Homo sapiens"
3	Remove	standard_type	"ratio," "fraction," "dose," "clast"
4	Remove	description	"patient," "nonrenal," "obese," "metabolite," "week" and "twice"

The unit was unified into mL/min/kg, and the original source for 94 records was manually checked to disclose body weight. A total of 54 out of 94 records were successfully corrected by listed averaged body weight in the literature. When there was an expression such as "healthy adult" or "healthy volunteer," 70 kg was used to correct the unit. Then, 209 compounds derived from ChEMBL were selected by checking the overlapped compounds with their molregno.

For literature (Varma et al. 2009[6], 2010[7], Ito et al.[8])-derived data, molregno was assigned to listed compounds in each dataset using structure information or compound name, then the CL_r and $f_{u,p}$ data were merged according to their molregno. Finally, 350 compounds were extracted.

A total of 209 and 350 CL_r values derived from ChEMBL and the literature (Varma et al. 2009[6], 2010[7], Ito et al.[8]) were merged into a single dataset. Overlaps were checked using molregno, structure information, and compound name. If overlapped compounds were identified, their average and standard deviation were calculated. If the standard deviation was more than 20% error, the original sources or the relevant interview forms (Common Technical Document for the registration of Pharmaceuticals) were checked manually. A total of 386 compounds remained after checking for overlap and 15 compounds were added by manual correction from CTD and the literature. CL_r greater than 0.001 was used to ensure a reliable clearance ratio. If a value of $f_{u,p}$ was not exit in the Dataset_CL_r, experimental $f_{u,p}$ values were searched in Watanabe et al.[4], the relevant Interview Forms, and in-house data. Ionization profiles were extracted from the ChEMBL database. Finally, Dataset_CL_r consisting of 401 compounds with CL_r, $f_{u,p}$, and structure information was generated.

1. Benet, L. Z.; Broccatelli, F.; Oprea, T. I., BDDCS applied to over 900 drugs. *AAPS J* **2011,** *13* (4), 519-47.

2. Hosey, M., C.; Chan, R.; Benet, Z., L., BDDCS Predictions, Self-Correcting Aspects of BDDCS Assignments, BDDCS Assignment Corrections, and Classification for more than 175 Additional Drugs. *AAPS J.* **2016,** *18* (1), 251-260.

3. Pharmapendium. https://www.elsevier.com/.

4. Watanabe, R.; Esaki, T.; Kawashima, H.; Natsume-Kitatani, Y.; Nagao, C.; Ohashi, R.; Mizuguchi, K., Predicting Fraction Unbound in Human Plasma from Chemical Structure: Improved Accuracy in the Low Value Ranges. *Mol Pharm* **2018,** *15* (11), 5302-5311.

5. Gaulton, A.; Bellis, L. J.; Bento, A. P.; Chambers, J.; Davies, M.; Hersey, A.; Light, Y.; McGlinchey, S.; Michalovich, D.; Al-Lazikani, B.; Overington, J. P., ChEMBL: a large-scale bioactivity database for drug discovery. *Nucleic Acids Res* **2012,** *40* (Database issue), D1100-7.

6. Varma, M. V.; Feng, B.; Obach, R. S.; Troutman, M. D.; Chupka, J.; Miller, H. R.; El-Kattan, A., Physicochemical determinants of human renal clearance. *J Med Chem* **2009,** *52* (15), 4844-52.

7. Varma, M. V.; Obach, R. S.; Rotter, C.; Miller, H. R.; Chang, G.; Steyn, S. J.; El-Kattan, A.; Troutman, M. D., Physicochemical space for optimum oral bioavailability: contribution of human intestinal absorption and first-pass elimination. *J Med Chem* **2010,** *53* (3), 1098-108.

8. Ito, S.; Ando, H.; Ose, A.; Kitamura, Y.; Ando, T.; Kusuhara, H.; Sugiyama, Y., Relationship between the urinary excretion mechanisms of drugs and their physicochemical properties. *J Pharm Sci* **2013,** *102* (9), 3294-301.

9. Shellenberger, M. K.; Groves, L.; Shah, J.; Novack, G. D., A controlled pharmacokinetic evaluation of tizanidine and baclofen at steady state. *Drug Metab Dispos* **1999,** *27* (2), 201-4.
10. Jaehde, U.; Sorgel, F.; Naber, K. G.; Zurcher, J.; Schunack, W., Distribution kinetics of enoxacin and its metabolite oxoenoxacin in excretory fluids of healthy volunteers. *Antimicrob Agents Chemother* **1995,** *39* (9), 2092-7.
11. DeSante, K. A.; Zeckel, M. L., Pharmacokinetic profile of loracarbef. *Am J Med* **1992,** *92* (6A), 16S-19S.
12. Majumdar, A. K.; Musson, D. G.; Birk, K. L.; Kitchen, C. J.; Holland, S.; McCrea, J.; Mistry, G.; Hesney, M.; Xi, L.; Li, S. X.; Haesen, R.; Blum, R. A.; Lins, R. L.; Greenberg, H.; Waldman, S.; Deutsch, P.; Rogers, J. D., Pharmacokinetics of ertapenem in healthy young volunteers. *Antimicrob Agents Chemother* **2002,** *46* (11), 3506-11.
13. Barriere, S. L., Pharmacology and pharmacokinetics of cefprozil. *Clin Infect Dis* **1992,** *14 Suppl 2*, S184-8; discussion S195-6.

Supplemental information 3. List of descriptors calculation failed

1	AATS7dv	41	AATSC7are	81	GATS7m	121	MAXsssNH	161	MAXaaSe	201	MINdsN	241	MINdssSe
2	AATS8dv	42	AATSC8are	82	GATS8m	122	MAXdsN	162	MAXdssSe	202	MINaaN	242	MINddssSe
3	AATS7d	43	AATSC7p	83	GATS7v	123	MAXaaN	163	MAXddssSe	203	MINsssN	243	MINsBr
4	AATS8d	44	AATSC8p	84	GATS8v	124	MAXsssN	164	MAXsBr	204	MINddsN	244	MINsSnH3
5	AATS7s	45	AATSC7i	85	GATS7se	125	MAXddsN	165	MAXsSnH3	205	MINaasN	245	MINssSnH2
6	AATS8s	46	AATSC8i	86	GATS8se	126	MAXaasN	166	MAXssSnH2	206	MINssssN	246	MINsssSnH
7	AATS7Z	47	MATS7c	87	GATS7pe	127	MAXssssN	167	MAXsssSnH	207	MINsOH	247	MINssssSn
8	AATS8Z	48	MATS8c	88	GATS8pe	128	MAXsOH	168	MAXssssSn	208	MINdO	248	MINsI
9	AATS7m	49	MATS7dv	89	GATS7are	129	MAXdO	169	MAXsI	209	MINssO	249	MINsPbH3
10	AATS8m	50	MATS8dv	90	GATS8are	130	MAXssO	170	MAXsPbH3	210	MINaaO	250	MINssPbH2
11	AATS7v	51	MATS7d	91	GATS7p	131	MAXaaO	171	MAXssPbH2	211	MINsF	251	MINsssPbH
12	AATS8v	52	MATS8d	92	GATS8p	132	MAXsF	172	MAXsssPbH	212	MINsSiH3	252	MINssssPb
13	AATS7se	53	MATS7s	93	GATS7i	133	MAXsSiH3	173	MAXssssPb	213	MINssSiH2	253	MDEC.11
14	AATS8se	54	MATS8s	94	GATS8i	134	MAXssSiH2	174	MINsLi	214	MINsssSiH	254	MDEC.12
15	AATS7pe	55	MATS7Z	95	MAXsLi	135	MAXsssSiH	175	MINssBe	215	MINssssSi	255	MDEC.13
16	AATS8pe	56	MATS8Z	96	MAXssBe	136	MAXssssSi	176	MINssssBe	216	MINsPH2	256	MDEC.14
17	AATS7are	57	MATS7m	97	MAXssssBe	137	MAXsPH2	177	MINssBH	217	MINssPH	257	MDEC.22
18	AATS8are	58	MATS8m	98	MAXssBH	138	MAXssPH	178	MINsssB	218	MINsssP	258	MDEC.24
19	AATS7p	59	MATS7v	99	MAXsssB	139	MAXsssP	179	MINssssB	219	MINdsssP	259	MDEC.34
20	AATS8p	60	MATS8v	100	MAXssssB	140	MAXdsssP	180	MINsCH3	220	MINsssssP	260	MDEC.44
21	AATS7i	61	MATS7se	101	MAXsCH3	141	MAXsssssP	181	MINdCH2	221	MINsSH	261	MDEO.11
22	AATS8i	62	MATS8se	102	MAXdCH2	142	MAXsSH	182	MINssCH2	222	MINdS	262	MDEO.12
23	AATSC7c	63	MATS7pe	103	MAXssCH2	143	MAXdS	183	MINtCH	223	MINssS	263	MDEO.22
24	AATSC8c	64	MATS8pe	104	MAXtCH	144	MAXssS	184	MINdsCH	224	MINaaS	264	MDEN.11
25	AATSC7dv	65	MATS7are	105	MAXdsCH	145	MAXaaS	185	MINaaCH	225	MINdssS	265	MDEN.12
26	AATSC8dv	66	MATS8are	106	MAXaaCH	146	MAXdssS	186	MINsssCH	226	MINddssS	266	MDEN.13

27	AATSC7d	67	MATS7p	107	MAXsssCH	147	MAXddssS	187	MINddC	227	MINsCl	267	MDEN.22
28	AATSC8d	68	MATS8p	108	MAXddC	148	MAXsCl	188	MINtsC	228	MINsGeH3	268	MDEN.23
29	AATSC7s	69	MATS7i	109	MAXtsC	149	MAXsGeH3	189	MINdssC	229	MINssGeH2	269	MDEN.33
30	AATSC8s	70	MATS8i	110	MAXdssC	150	MAXssGeH2	190	MINaasC	230	MINsssGeH		
31	AATSC7Z	71	GATS7c	111	MAXaasC	151	MAXsssGeH	191	MINaaaC	231	MINssssGe		
32	AATSC8Z	72	GATS8c	112	MAXaaaC	152	MAXssssGe	192	MINssssC	232	MINsAsH2		
33	AATSC7m	73	GATS7dv	113	MAXssssC	153	MAXsAsH2	193	MINsNH3	233	MINssAsH		
34	AATSC8m	74	GATS8dv	114	MAXsNH3	154	MAXssAsH	194	MINsNH2	234	MINsssAs		
35	AATSC7v	75	GATS7d	115	MAXsNH2	155	MAXsssAs	195	MINssNH2	235	MINsssdAs		
36	AATSC8v	76	GATS8d	116	MAXssNH2	156	MAXsssdAs	196	MINdNH	236	MINsssssAs		
37	AATSC7se	77	GATS7s	117	MAXdNH	157	MAXsssssAs	197	MINssNH	237	MINsSeH		
38	AATSC8se	78	GATS8s	118	MAXssNH	158	MAXsSeH	198	MINaaNH	238	MINdSe		
39	AATSC7pe	79	GATS7Z	119	MAXaaNH	159	MAXdSe	199	MINtN	239	MINssSe		
40	AATSC8pe	80	GATS8Z	120	MAXtN	160	MAXssSe	200	MINsssNH	240	MINaaSe		

Supplemental Information 4

Scheme S1. Common process of model construction

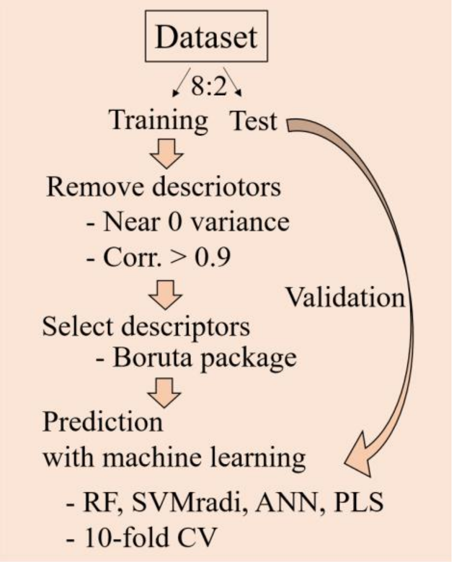

Scheme S2. Overview of prediction model construction for CL_r

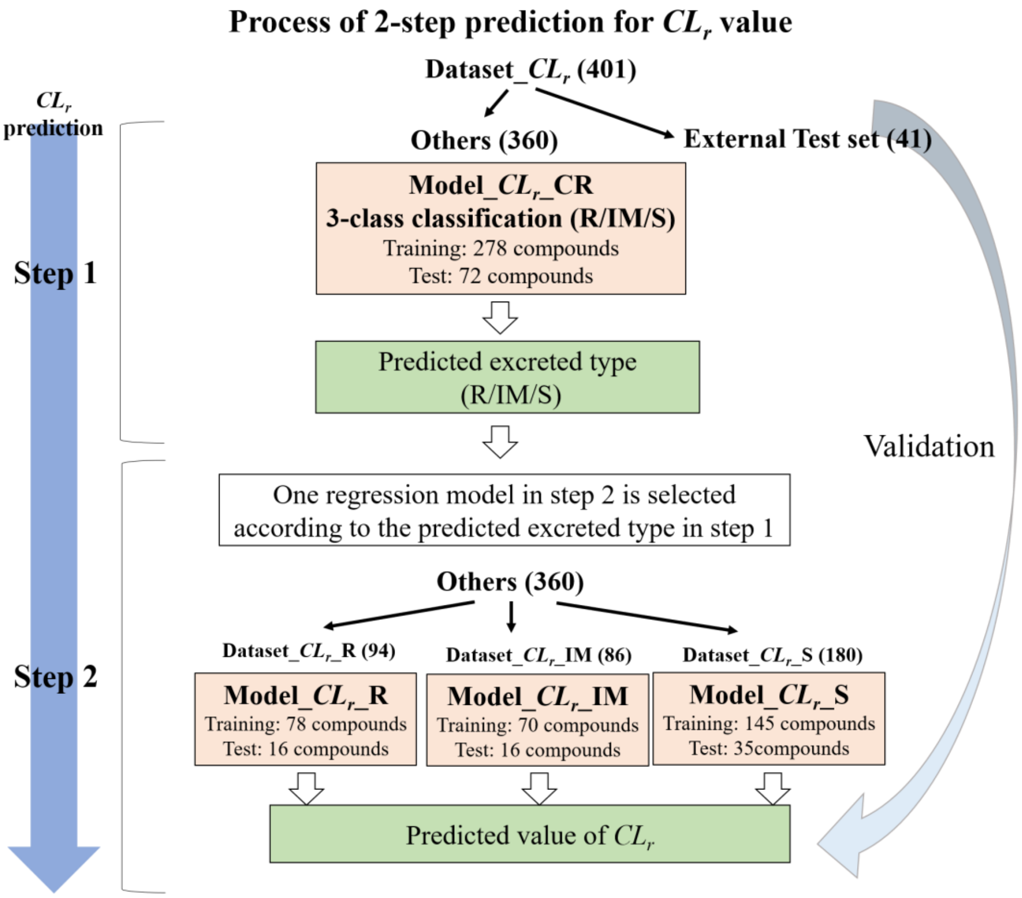

Tables

Table S1. Statistical results of the binary classification model for f_e with or without $f_{u,p}$ value as a descriptor.

Type[a]	Tr or Te	Method	Split 1	Split 2	Split 3	Split 4	Av.	SD	p-value[b]
Models without $f_{u,p}$ (Model_f_e)	Training	RF	0.59	0.50	0.47	0.53	0.52	0.05	-
		SVM	0.59	0.45	0.46	0.54	0.51	0.06	-
		ANN	0.48	0.45	0.49	0.51	0.48	0.02	-
		PLS	0.52	0.46	0.44	0.52	0.48	0.03	-
	Test	RF	0.30	0.47	0.46	0.48	0.43	0.08	-
		SVM	0.38	0.42	0.43	0.30	0.38	0.05	-
		ANN	0.45	0.39	0.31	0.31	0.36	0.06	-
		PLS	0.40	0.44	0.40	0.23	0.37	0.08	-
Models with observed $f_{u,p}$	Training	RF	0.58	0.53 [a]	0.52	0.54	0.54	0.02	0.271756
		SVM	0.54	0.49	0.45	0.52	0.50	0.03	0.607563
		ANN	0.49	0.46	0.45	0.47	0.47	0.01	0.289854
		PLS	0.53	0.46	0.48	0.53	0.50	0.03	0.151834
	Test	RF	0.25	0.49[a]	0.49	0.52	0.44	0.11	0.691126
		SVM	0.39	0.42	0.38	0.32	0.38	0.04	0.676281
		ANN	0.34	0.42	0.38	0.41	0.39	0.03	0.656068
		PLS	0.47	0.44	0.35	0.33	0.40	0.06	0.399535
Models with predicted $f_{u,p}$	Training	RF	0.60	0.54	0.53[a]	0.56	0.56	0.03	0.069243
		SVM	0.57	0.51	0.46	0.49	0.51	0.04	0.984526
		ANN	0.53	0.49	0.50	0.50	0.50	0.02	0.151909
		PLS	0.55	0.52	0.53	0.48	0.52	0.02	0.291087
	Test	RF	0.32	0.43	0.51[a]	0.50	0.44	0.08	0.625774
		SVM	0.38	0.45	0.43	0.30	0.39	0.06	0.391002
		ANN	0.37	0.42	0.24	0.32	0.34	0.07	0.41385
		PLS	0.54	0.51	0.44	0.39	0.47	0.06	0.027711

[a]Three types of descriptor sets were utilized to generate the models: 1) 6974 descriptors; 2) 6974 descriptors + predicted $f_{u,p}$, and 3) 6974 descriptors + observed $f_{u,p}$.

[b]Prediction models of f_e were constructed using each descriptor set and p-value was calculated using the paired t-test between the Kappa of Model_f_e and those of the prediction model generated with descriptor set of 2) and 3) in the training and test set with four different random splits, respectively.

Table S2. The important descriptors and descriptions in the binary classification model for f_e prediction

Without $f_{u,p}$ (Model_f_e)	With pred. $f_{u,p}$	With obs. $f_{u,p}$	Descriptor	Description
1	1	1	SLogP	Wildman-Crippen LogP
-	2	-	fup_pred_log	Predicted fu,p in logarithmic scale
-	-	2	fup_obs_log	Observed fu,p in logarithmic scale
10	10	-	AATS3i	Averaged Moreau-Broto autocorrelation of lag 3 weighted by ionization potential
5	4	-	AATSC1c	Broto autocorrelation of lag 1 weighted by gasteiger charge
3	-	-	ATS8i	Moreau-Broto autocorrelation of lag 8 weighted by ionization potential
-	9	9	ATSC1dv	Centered Moreau-Broto autocorrelation of lag 1 weighted by valence electrons
-	7	-	AXp.4dv	4-ordered averaged Chi path weighted by sigma electrons
-	-	8	BCUTdv.1h	First highest eigenvalue of Burden matrix weighted by valence electrons
4	5	4	ETA_dEpsilon_D	ETA delta epsilon (type: D)
-	-	6	GATS1se	Geary coefficient of lag 1 weighted by Sanderson electronegativity
7	3	-	GATS4are	Geary coefficient of lag 4 weighted by Allred-Rochow electronegativity
8	-	-	GATS4s	Geary coefficient of lag 4 weighted by intrinsic state
-	-	7	MATS4Z	Moran coefficient of lag 4 weighted by atomic number
9	-	5	nAcid	Acidic group count
-	8	-	nHBDon	Number of hydrogen bond donor
6	-	-	Xc.5d	5-ordered Chi cluster weighted by sigma electrons
2	6	3	Xch.5dv	5-ordered Chi chain weighted by valence electrons

ETA, Extended topochemical atom; pred., predicted; obs., observed

Table S3. Statistical results of the single regression classification models for CL_r prediction by each of the six machine learning models.

Model	Parameter	RF[a]	SVM radi[a]	SVM lin[a]	ANN[a]	k-NN[a]	PLS[a]
without $f_{u,p}$	r^2	0.32	0.33	0.21	0.16	0.18	0.26
	RMSE	0.76	0.77	0.86	0.93	0.89	0.79
with predicted $f_{u,p}$	r^2	0.37	0.39	0.34	0.33	0.23	0.28
	RMSE	0.73	0.73	0.75	0.80	0.85	0.78
with observed $f_{u,p}$	r^2	0.40	0.35	0.27	0.34	0.25	0.33
	RMSE	0.71	0.76	0.81	0.76	0.83	0.75

[a]: RF, Random Forest; SVM radi or lin, Support Vector Machine with radial and linear basis functions; ANN, artificial neural network; k-NN, k-nearest neighbors; PLS, partial least squares

Table S4. The important descriptors in regression models generated using predicted $f_{u,p}$ values for CL_r prediction

Model_CL_r_R		Model_ CL_r _IM		Model_ CL_r_S	
Rank	Descriptor	Rank	Descriptor	Rank	Descriptor
1	fup_pred_log	1	fup_pred_log	1	fup_pred_log
2	GATS2are	2	SlogP_VSA6	2	nAHRing
3	MATS3i	3	AATSC2v	3	Xc.6dv
4	EState_VSA6	4	JGI2	4	NaaCH
5	AATS1i	5	AATS5p	5	C2SP2

Table S5. Description of important descriptors in regression models for CL_r prediction

Descriptor	Description
AATS1i	Averaged Moreau-Broto autocorrelation of lag 1 weighted by ionization potential
AATS5p	Broto autocorrelation of lag 5 weighted by Pauling EN
AATSC2v	Broto autocorrelation of lag 2 weighted by vdw volume
C2SP2	SP2 carbon bound to 2 other carbons
EState_VSA6	EState VSA Descriptor 6
fup_pred_log	Predicted *fu,p* in logarithmic scale
GATS2are	Geary coefficient of lag 2 weighted by Allred-Rocow EN
nAHRing	Aromatic hetero ring count
SMR_VSA6	MOE MR VSA Descriptor 6
MATS3i	Moran coefficient of lag 3 weighted by ionization potential
SlogP_VSA6	MOE logP VSA Descriptor 6 (0.15 <= x < 0.20)
JGI2	2-ordered mean topological charge
Xc.6dv	6-ordered Chi cluster weighted by valence electrons

vdw; van der Waals, VSA; van der Waals surface area, MR; molecular refractivity, EN; electronegativity

Table S6. Raw statistical data of Model_CR

		Sensitivity	Specificity	Pos.Pred.Value	Neg.Pred.Value	Balanced.Accuracy
**RF**	**Class: IM**	**0.29**	**0.87**	**0.42**	**0.80**	**0.58**
	**Class: R**	**0.56**	**0.85**	**0.56**	**0.85**	**0.70**
	**Class: S**	**0.75**	**0.60**	**0.66**	**0.70**	**0.68**
SVM	Class: IM	0.12	0.89	0.25	0.76	0.50
	Class: R	0.56	0.83	0.53	0.85	0.69
	Class: S	0.69	0.46	0.57	0.59	0.58
ANN	Class: IM	0.41	0.78	0.37	0.81	0.59
	Class: R	0.56	0.81	0.50	0.84	0.68
	Class: S	0.47	0.57	0.53	0.51	0.52
PLS	Class: IM	0.18	0.91	0.38	0.78	0.54
	Class: R	0.50	0.87	0.56	0.84	0.68
	Class: S	0.75	0.43	0.57	0.63	0.59

Table S7. Statistical result of three-class classification models to predict CR type (R/IM/S). p-values were calculated using the paired t-test between Kappa of the model with or without $f_{u,p}$ in five random splits

			Split 1	Split 2	Split 3	Split 4	Split 5	Av.	SD	p-value
Without $f_{u,p}$	Training	RF	0.33	0.25	0.29	0.34	0.31	0.30	0.03	
		SVM	0.26	0.27	0.35	0.36	0.33	0.31	0.04	
		ANN	0.25	0.21	0.30	0.32	0.28	0.27	0.04	
		PLS	0.24	0.25	0.30	0.27	0.25	0.26	0.02	
	Test	RF	0.20	0.21	0.26	0.23	0.24	0.23	0.02	
		SVM	0.24	0.17	0.17	0.31	0.28	0.23	0.05	
		ANN	0.10	0.22	0.01	0.14	0.17	0.13	0.07	
		PLS	0.13	0.11	0.10	0.18	0.23	0.15	0.05	
With observed $f_{u,p}$	Training	RF	0.34	0.33	0.38	0.31	0.36	0.34	0.02	0.11
		SVM	0.28	0.29	0.38	0.37	0.30	0.32	0.04	0.45
		ANN	0.27	0.27	0.34	0.34	0.36	0.32	0.04	0.02
		PLS	0.25	0.23	0.38	0.33	0.19	0.28	0.07	0.57
	Test	RF	0.22	0.20	0.30	0.29	0.36	0.27	0.06	0.13
		SVM	0.22	0.22	0.18	0.27	0.18	0.22	0.03	0.49
		ANN	0.20	0.11	0.19	0.19	0.33	0.20	0.07	0.24
		PLS	0.10	0.22	0.08	0.16	0.18	0.15	0.05	0.96
With predicted $f_{u,p}$	Training	RF	0.25	0.26	0.31	0.27	0.31	0.28	0.03	0.34
		SVM	0.26	0.25	0.36	0.31	0.33	0.30	0.04	0.33
		ANN	0.24	0.28	0.27	0.27	0.25	0.26	0.01	0.67
		PLS	0.25	0.23	0.33	0.30	0.27	0.27	0.03	0.15
	Test	RF	0.19	0.22	0.28	0.18	0.21	0.21	0.03	0.33
		SVM	0.21	0.15	0.10	0.24	0.28	0.19	0.07	0.04
		ANN	0.16	0.15	0.17	0.08	0.12	0.13	0.03	0.95
		PLS	0.20	0.19	0.11	0.15	0.17	0.16	0.03	0.65

Table S8. Fold error of predicted CL_r by the two-step prediction system with or without $f_{u,p}$

Descriptor	Fold Error	All
Without $f_{u,p}$	within 2-fold	26.8
	within 3-fold	48.8
With observed $f_{u,p}$	within 2-fold	41.5
	within 3-fold	56.1
With predicted $f_{u,p}$	within 2-fold	39.0
	within 3-fold	43.9

Figures.

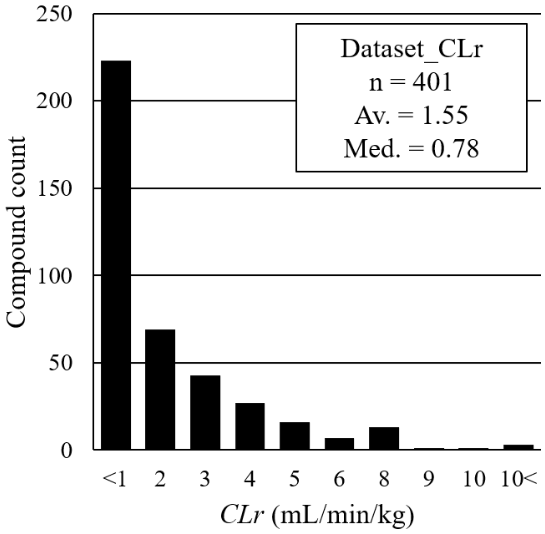

Figure S1. Distribution of CL_r in linear scale in Dataset_CL_r consisting of 401 compounds. Average and median are shown in the top-right.

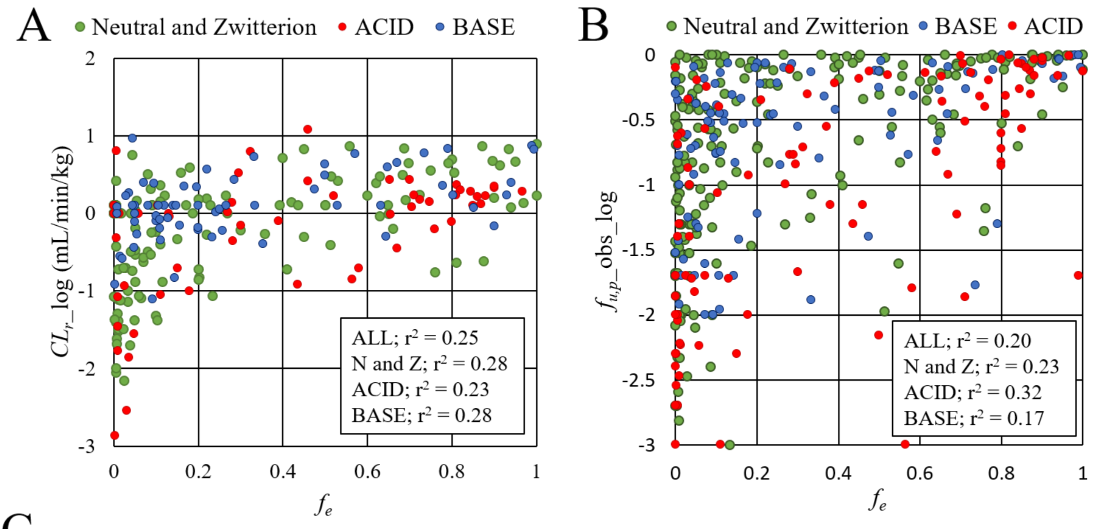

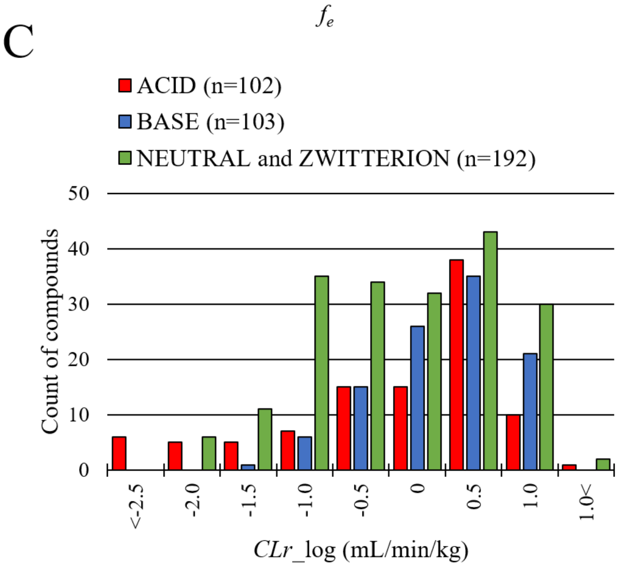

	CLr (mL/min/kg)	
	Av.	Median
ALL	1.55	0.78
ACID	1.38	0.93
BASE	1.89	1.24
NEUTRAL and ZWITTERION	1.49	0.47

Figure S2. (A) Relationship between f_e and CL_r in 220 common compounds in Dataset_ f_e and Datasaet_CL_r. Acids are shown as red circles, bases with blue, neutrals and zwitterions are green. (B) Relationship between f_e and $f_{u,p}$. (C) Plot of CL_r versus compound counts depending on ionization properties (102 acids, 103 bases, and 192 neutrals/zwitterions).

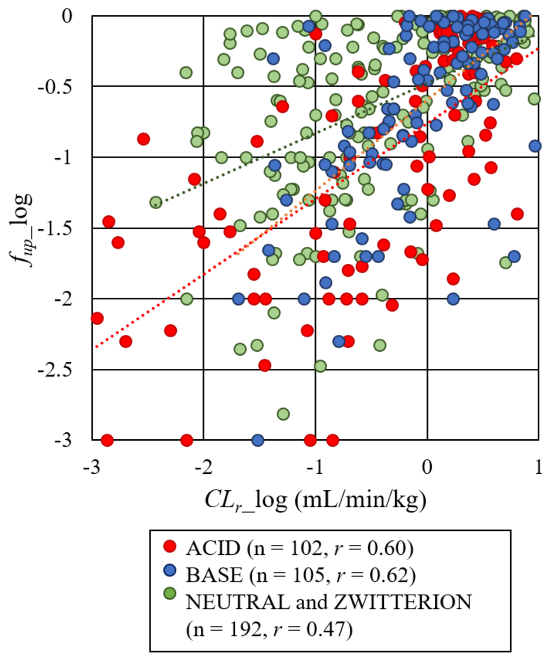

Figure S3. Plot of the relationship between $f_{u,p}$ and CL_r when the dataset was sub-categorized by ionization property (102, 105, and 192 compounds in acid, base, neutral, and zwitterion, respectively).

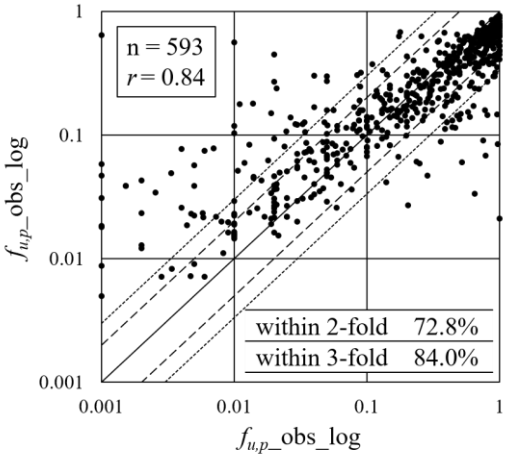

Figure S4. Correlation between predicted and observed $f_{u,p}$ values (r = 0.84) in 593 compounds of Dataset_f_e and Dataset_CL_r. A total of 72.8% and 84.0% of compounds fell within 2- and 3-fold error. Dotted line: 3- or 1/3-fold; dashed line: 2- or 1/2-fold; solid line: line of unity (x = y).

ID	Compound name	pred_CRclass	observed_CRclass
5	sparfloxacin	S	R
18	methylprednisolone	R	IM
20	trazodone	S	R
37	prednisolone	R	S
124	atropine	R	S
125	bupivacaine	R	IM
169	lamotrigine	S	R
261	2-hydroxyimipramine	R	IM
314	minocycline	IM	R
351	tetracycline	IM	R
356	domperidone	S	IM
391	canagliflozin	S	IM

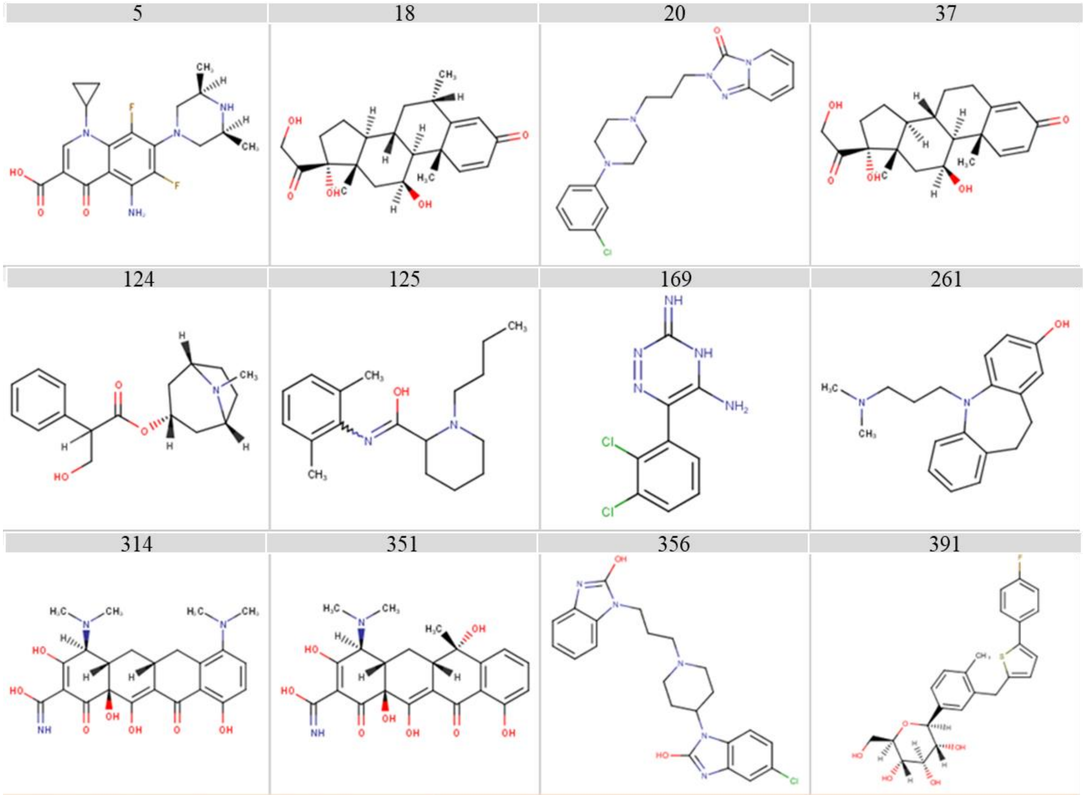

Figure S5. List of the compounds that were miss-classified in Model_CL_r_CR and did not fall into within 3-fold errors. Upper; List of the compounds. Lower; Structures of the compounds.
